# Supplementary material for: A novel class of inhibitors that disrupts the stability of integrin heterodimers identified by CRISPR-tiling-instructed genetic screens
Source: Nat Struct Mol Biol. 2024 Feb 5;31(3):465–75. doi: 10.1038/s41594-024-01211-y (PMC10948361; doi:10.1038/s41594-024-01211-y)
Supplement: Supplementary file 1 — Supplementary Figs. 1–4. [file 41594_2024_1211_MOESM1_ESM.pdf]

nature structural & molecular biology

Article https://doi.org/10.1038/s41594-024-01211-y

A novel class of inhibitors that disrupts the stability of integrin heterodimers identified by CRISPR-tiling-instructed genetic screens

In the format provided by the authors and unedited

Suppl Figure 1

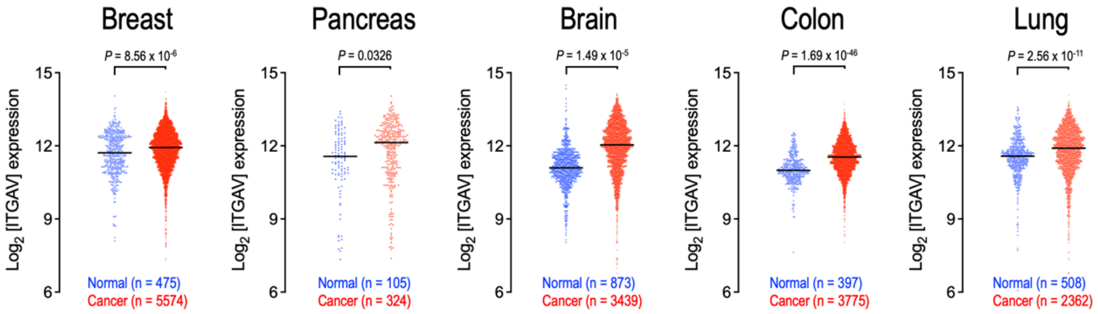

Supplementary Fig. 1. Expression of ITGAV in normal and cancer samples.
ITGAV is overexpressed in multiple cancer types, including the ones tested in our cell surface proteome CRISPR screens, i.e., the breast, pancreas, brain, colon, and lung cancers. Data source: GENT2 database (http://gent2.appex.kr/gent2/), which collected gene expression data from > 68,000 normal and cancer samples. *P* value by two-sided Student’s t-test.

Suppl Figure 2

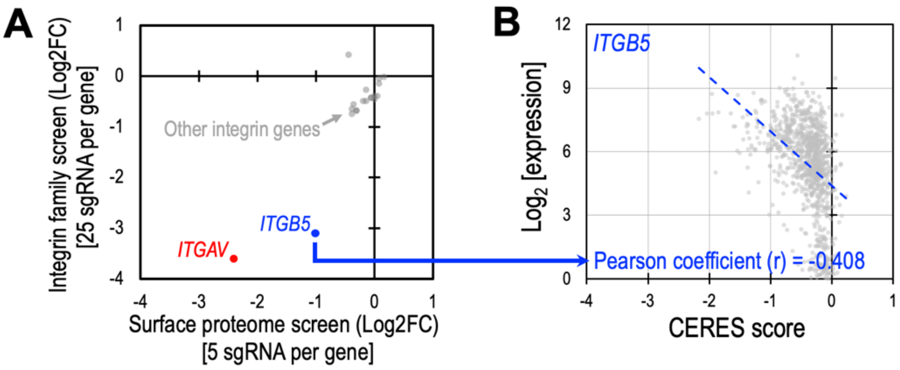

Supplementary Fig. 2. Integrin family CRISPR screens identify ITGB5 as an essential β integrin.
(A) In the surface proteome CRISPR screens (x-axis; ~580 genes with 5 sgRNAs/gene), we identified ITGAV (red) as the top essential surface gene. However, which β integrin to serve as the critical partner of ITGAV was not clearly revealed. We, therefore, performed the 2nd screen focusing only on the 26 integrin family genes with 25 sgRNAs/gene (y-axis). The increased sgRNA number per gene allowed the identification of ITGB5 as the top essential β integrin (blue). (B) Similar to *ITGAV* (see Extended Data Fig. 2D, left panel), the cancer cell dependency on *ITGB5* is correlated with its expression (source: https://depmap.org/portal/; BROAD Institute), highlighting the role of integrin αVβ5 in cancer cell maintenance. Notably, while the 1st screen didn't observe ITGB5 within the leading-edge essential genes, re-evaluating of this surface proteome screen revealed that ITGB5 remains the 2nd most essential integrin (Panel A).

Suppl Figure 3

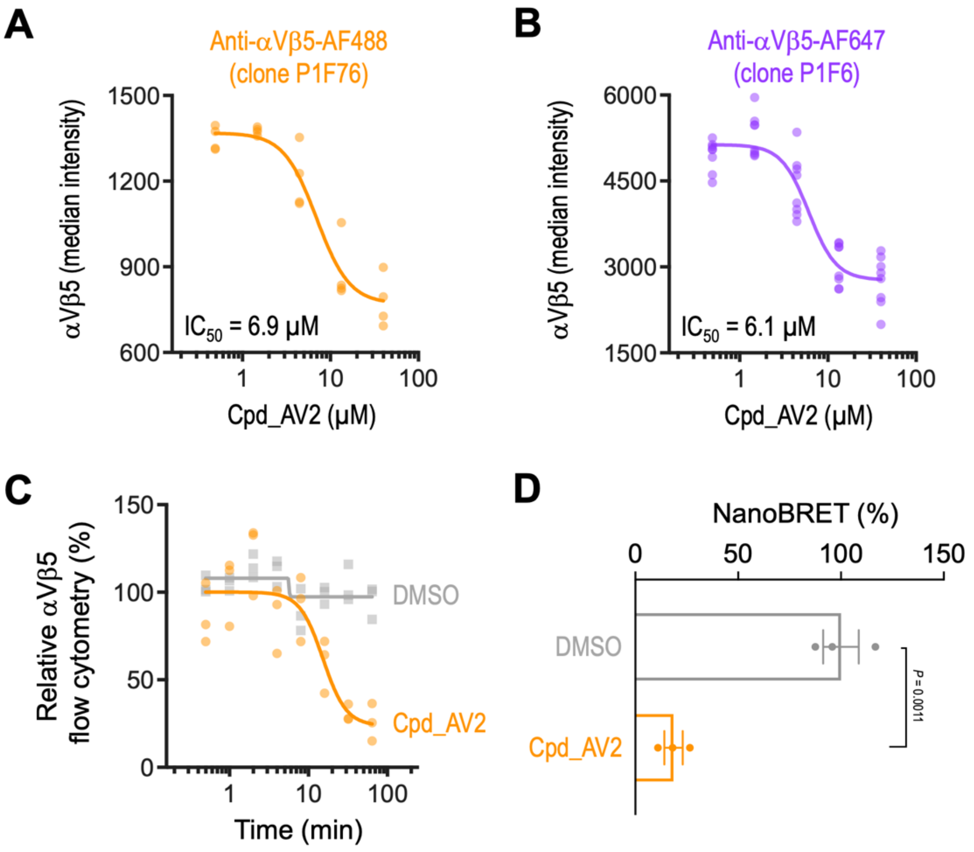

Supplementary Fig. 3. Validation of the effects of Cpd_AV2 on integrin αVβ5 heterodimer.
The capacity of Cpd_AV2 to disrupt cell surface integrin αVβ5 heterodimer is validated via two monoclonal integrin αVβ5 antibodies (A) clone P1F76 (sc-13588, Santa Cruz; data presented in Fig. 5E and F; n = 4 for each condition) and (B) clone P1F6 (920005, Biolegend; n = 8 for each condition). (C) To observe the dynamic changes of αVβ5 heterodimer level upon Cpd_AV2 treatment, we monitored the amount of αVβ5 heterodimer on the cell surface (clone P1F76 antibody) from 0.5 min to 64 min after 40 μM Cpd_AV2 treatment (n = 3 for each group). These results indicated that a 1-hour Cpd_AV2 incubation is sufficient to disrupt the αVβ5 heterodimers. (D) Integrin αVβ5 NanoBRET assay validates the capacity of 1-hour Cpd_AV2 incubation to destabilize integrin αVβ5 heterodimer (n = 3 for each group). Data are represented as mean ± SEM. *P* value by two-sided Student's t-test.

Suppl Figure 4

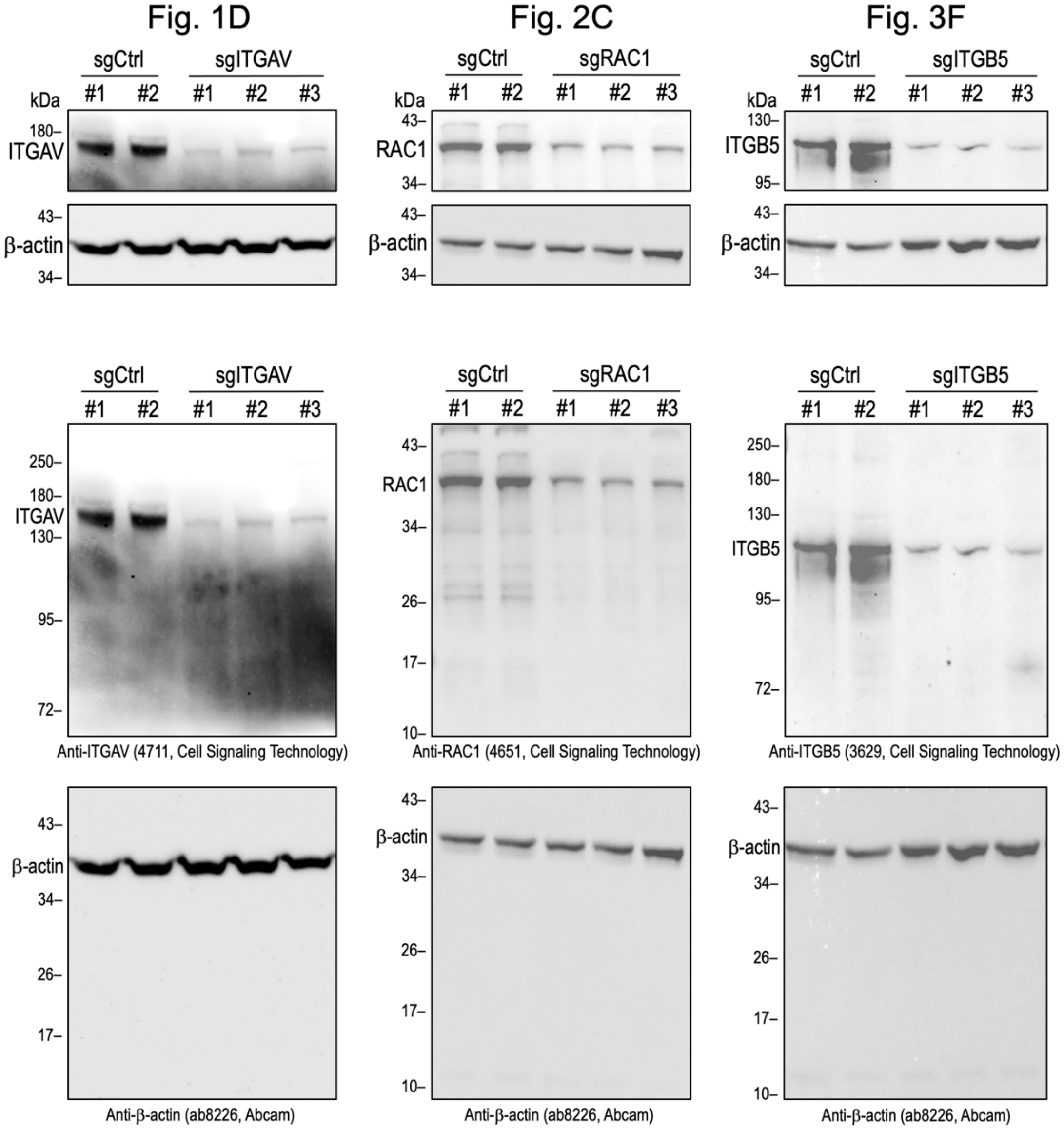

Supplementary Fig. 4. Original Western blot images.

The uncropped Western blot images for (left) Fig. 1D, (mid) Fig. 2C, and (right) Fig. 3F.
