# Supplementary material for: A novel class of inhibitors that disrupts the stability of integrin heterodimers identified by CRISPR-tiling-instructed genetic screens
Source: Nat Struct Mol Biol. 2024 Feb 5;31(3):465–75. doi: 10.1038/s41594-024-01211-y (PMC10948361; doi:10.1038/s41594-024-01211-y)
Supplement: Supplementary file 2 — Reporting Summary [file 41594_2024_1211_MOESM2_ESM.pdf]

nature research

Corresponding author(s): Chun-Wei David Chen

Last updated by author(s): 11/4/2023

Reporting Summary

Nature Research wishes to improve the reproducibility of the work that we publish. This form provides structure for consistency and transparency in reporting. For further information on Nature Research policies, see our Editorial Policies and the Editorial Policy Checklist.

Statistics

For all statistical analyses, confirm that the following items are present in the figure legend, table legend, main text, or Methods section.

n/a	Confirmed	
☐	☒	The exact sample size (*n*) for each experimental group/condition, given as a discrete number and unit of measurement
☐	☒	A statement on whether measurements were taken from distinct samples or whether the same sample was measured repeatedly
☐	☒	The statistical test(s) used AND whether they are one- or two-sided<br>*Only common tests should be described solely by name; describe more complex techniques in the Methods section.*
☒	☐	A description of all covariates tested
☒	☐	A description of any assumptions or corrections, such as tests of normality and adjustment for multiple comparisons
☐	☒	A full description of the statistical parameters including central tendency (e.g. means) or other basic estimates (e.g. regression coefficient) AND variation (e.g. standard deviation) or associated estimates of uncertainty (e.g. confidence intervals)
☐	☒	For null hypothesis testing, the test statistic (e.g. *F*, *t*, *r*) with confidence intervals, effect sizes, degrees of freedom and *P* value noted<br>*Give P values as exact values whenever suitable.*
☒	☐	For Bayesian analysis, information on the choice of priors and Markov chain Monte Carlo settings
☒	☐	For hierarchical and complex designs, identification of the appropriate level for tests and full reporting of outcomes
☐	☒	Estimates of effect sizes (e.g. Cohen's *d*, Pearson's *r*), indicating how they were calculated

Our web collection on statistics for biologists contains articles on many of the points above.

Software and code

Policy information about availability of computer code

Data collection	Sequencing data was collected on NextSeq 550 and NovaSeq 6000 (Illumina)
Data analysis	The computational codes/tool packages used in this study include Genetic Perturbation Platform (BROAD Institute), Bowtie2 (Johns Hopkins University), UCSF Chimera 1.15 (UC San Francisco), Attune NxT v3.1.2 (ThermoFisher), GSEA v4.1.0 (UC San Diego and BROAD Institute), FASTQC v0.11.8, Burrows-Wheeler Aligner v0.7.17, MACS2 v2.1.1, Samtools v1.10, STAR v2.6.1d, featureCounts v1.6.4, edgeR v4.0.1, deepTools v3.5.1, IGV 2.14.0 (BROAD Institute), PyMOL v2.0.4 (Schrödinger, LLC), PDB2PQR server, AutoDockTools, AutoSite, Open Babel v2.4.1, PyRx v0.9.7, AutoDock Vina v1.1.2, Raccoon2, JTSA (https://paulsbond.co.uk/jtsa), Bio-Rad ChemiDoc MP (Bio-Rad), MAGeCK v0.5.9.2. IC50 and two-sided Student's t-test were performed using Prism 9 (GraphPad).

For manuscripts utilizing custom algorithms or software that are central to the research but not yet described in published literature, software must be made available to editors and reviewers. We strongly encourage code deposition in a community repository (e.g. GitHub). See the Nature Research guidelines for submitting code & software for further information.

Data

Policy information about availability of data

All manuscripts must include a data availability statement. This statement should provide the following information, where applicable:

- Accession codes, unique identifiers, or web links for publicly available datasets
- A list of figures that have associated raw data
- A description of any restrictions on data availability

The RNA-seq data generated in this study are available via Gene Expression Omnibus (GEO) under accession GSE231339. All the data supporting the findings of this study are included in this article and its Supplementary Information. Three-dimensional protein structure (PDB ID: 3IJE) was obtained from the Research Collaboratory for Structural Bioinformatics Protein Data Bank (RCSB PDB; https://www.rcsb.org). ITGAV expression data in breast, pancreas, brain, colon, and lung

cancers was obtained from Gene Expression database of Normal and Tumor tissues (GENT2 database: http://gent2.appex.kr/gent2/). Additional data that support the findings of this study are provided in the Supplementary Information.

Field-specific reporting

Please select the one below that is the best fit for your research. If you are not sure, read the appropriate sections before making your selection.

☒ Life sciences ☐ Behavioural & social sciences ☐ Ecological, evolutionary & environmental sciences

For a reference copy of the document with all sections, see nature.com/documents/nr-reporting-summary-flat.pdf

Life sciences study design

All studies must disclose on these points even when the disclosure is negative.

Sample size	Fig. 1A-C: total 2973 sgRNAs library screen performed in 5 cell line models. n = 3 was chosen to allow two-sided Student's t-test.<br>Fig. 1D: 2 independent sgCtrl sequences and 3 independent sgITGAV sequences. Sample size was chosen based on the available independent sgRNA numbers.<br>Fig. 1E: 2 independent sgCtrl sequences and 3 independent sgITGAV sequences. n = 3 for each sgRNA group was chosen to allow two-sided Student's t-test.<br>Fig. 1F,G: n = 3 for each sgRNA group was chosen to allow two-sided Student's t-test.<br>Fig. 1H: n = 927 patients for each ITGAV(high) and ITGAV(low) groups. Sample size was chosen based on the total available data from the GEPIA database.<br>Fig. 2A: 17110 genes tested in 769 cell models. Sample size was chosen based on the total available data from the DepMap database.<br>Fig. 2B: n = 3 for each sgRNA group was chosen to allow GSEA analysis.<br>Fig. 2C: 2 independent sgCtrl sequences and 3 independent sgRAC1 sequences. Sample size was chosen based on the available independent sgRNA numbers.<br>Fig. 2D: 2 independent sgCtrl sequences and 3 independent sgRAC1 sequences. n = 3 for each sgRNA group was chosen to allow two-sided Student's t-test.<br>Fig. 2E,F: n = 3 for each sgRNA group was chosen to allow two-sided Student's t-test.<br>Fig. 2H: sgCtrl (n = 30), sgITGAV (n = 42), and sgRAC1 (n = 33). Sample size was chosen based on available cell number for each sgRNA group.<br>Fig. 3B-D: total 712 sgRNAs library screen performed in 2 cell line models. n = 3 was chosen to allow two-sided Student's t-test.<br>Fig. 3E: 2 independent sgCtrl sequences and 3 independent sgRNA sequences for each ITGB gene. n = 3 for each sgRNA group was chosen to allow two-sided Student's t-test.<br>Fig. 3F: 2 independent sgCtrl sequences and 3 independent sgITGB5 sequences. Sample size was chosen based on the available independent sgRNA numbers.<br>Fig. 3G,H: n = 3 for each sgRNA group was chosen to allow two-sided Student's t-test.<br>Fig. 3I: 17110 genes tested in 769 cell models. Sample size was chosen based on the total available data from the DepMap database.<br>Fig. 4A,B: total 412 sgRNAs library screen performed in MDA231-Cas9 cells. n = 3 was chosen to allow two-sided Student's t-test.<br>Fig. 4F: n = 3 for each ITGAV cDNA group was chosen to allow two-sided Student's t-test.<br>Fig. 5B: total 128562 compounds. Sample size was chosen based on the total available compounds collected by the NCI/DTP Open Chemicals Repository.<br>Fig. 5C: total 500 compounds with the top binding energy were chosen for CellTiterGlo and CCK8 assays. n = 3 for each condition was chosen to allow two-sided Student's t-test.<br>Fig. 5F: n = 4 in this experiment based on the available cell cultures. A minimum of n = 3 is required for IC50 test.<br>Fig. 5G,H: n = 3 for each treatment group was chosen to allow two-sided Student's t-test.<br>Fig. 5J: Control (n = 30) and Cpd_AV2 (n = 33). Sample size was chosen based on available cell number for each treatment group.<br>Fig. 6A: Representative gel of 2 independent protein purification experiments.<br>Fig. 6B: Vehicle (724 data points) and Cpd_AV2 (724 data points) was chosen based on the available data from 25°C to 80°C.<br>Fig. 6D: n = 3 for each treatment group was chosen to allow two-sided Student's t-test.<br>Fig. 6E: n = 3 for each treatment group was chosen to allow two-sided Student's t-test and IC50 test.<br>Fig. 6F: n = 3 for each treatment group was chosen to allow IC50 test.
Data exclusions	No data point was excluded.
Replication	For Fig. 1A-C, 3B,C and 4A,B, triplicated library screens were performed. For other experiments, a minimum of 2 independent experiments were performed and all attempts at replication were successful.
Randomization	In cell culture experiment, an initial cell culture was split into individual cultures randomly with an equal seeding density. Each culture received a sgRNA, cDNA, CRISPR library, or compounds without predetermination.
Blinding	The same group of researchers designed, operated, and analyzed the experiments. Therefore, they were aware of the treatment conditions while executing and analyzing the experiments.

Reporting for specific materials, systems and methods

We require information from authors about some types of materials, experimental systems and methods used in many studies. Here, indicate whether each material, system or method listed is relevant to your study. If you are not sure if a list item applies to your research, read the appropriate section before selecting a response.

Materials & experimental systems

n/a	Involved in the study	
☐	☒	Antibodies
☐	☒	Eukaryotic cell lines
☒	☐	Palaeontology and archaeology
☒	☐	Animals and other organisms
☒	☐	Human research participants
☒	☐	Clinical data
☒	☐	Dual use research of concern

Methods

n/a	Involved in the study	
☒	☐	ChIP-seq
☐	☒	Flow cytometry
☒	☐	MRI-based neuroimaging

Antibodies

Antibodies used

Western blot: primary antibodies against ITGAV (4711, Cell Signaling Technology, 1:1000), ITGB5 (3629, Cell Signaling Technology; 1:1000), RAC1 (4651, Cell Signaling Technology; 1:1000), and beta-actin (ab8226, Abcam; 1:5000) at 4°C overnight. After washing, the membranes were incubated with HRP-conjugated goat anti-mouse (31430, Invitrogen; 1:10,000) or goat anti-rabbit (31460, Invitrogen; 1:10,000) IgG antibodies at room temperature for 1 hour. The chemiluminescent signals were detected using a ChemiDoc imaging system (Bio-Rad). The cell surface integrin αVβ5 was recognized by a mouse monoclonal anti-human αVβ5 antibody (clone P1F76; sc-13588, Santa Cruz Biotech; 1:200) and stained by AF488-conjugated donkey anti-mouse IgG (ab150105, Abcam) secondary antibody.

Validation

1. The specificity of anti-human-ITGAV (4711, Cell Signaling Technology, 1:1000), anti-human-ITGB5 (3629, Cell Signaling Technology; 1:1000), anti-human-RAC1 (4651, Cell Signaling Technology; 1:1000) to their target proteins was confirmed by Western blots with CRISPR depletion of the targeted protein in human cells as shown in Fig. 1D, 2C, 3F, and Suppl. Fig. 4.
2. The specificity of anti-human beta-actin (ab8226, Abcam; 1:5000) was evaluated by Western blot of human cell samples and observed a single band at the expected molecular weight (~40 kDa) as shown in Suppl. Fig. 4.
3. HRP-conjugated goat anti-mouse (31430, Invitrogen; 1:10,000) and goat anti-rabbit (31460, Invitrogen; 1:10,000) IgG secondary antibodies have been used in our lab for multiple projects, including the detection of protein IP and co-IP. These secondary antibodies have shown high specificity to only detect the primary antibodies from their targeted species (mouse, rabbit).
4. The specificity of monoclonal anti-human αVβ5 antibody (clone P1F76; sc-13588, Santa Cruz Biotech; 1:200) to cell surface integrin αVβ5 (stained by AF488-conjugated donkey anti-mouse IgG; ab150105, Abcam) was confirmed by flow cytometry of human cells with sgCtrl vs. sgITGAV as shown in Fig. 5E. Another mouse monoclonal anti-human αVβ5 antibody (clone P1F6; 920005, Biolegend; AF647-conjugated) was used to validate the αVβ5 flow cytometry results.

Eukaryotic cell lines

Policy information about cell lines

Cell line source(s)

HEK293, PANC1, and SW620 cells were obtained from the American Type Culture Collection (ATCC). MDA231 (i.e., MDA-MB-231) cells were obtained from Dr. Mingye Feng (City of Hope Cancer Center; original commercial source: ATCC). H661 cells were obtained from Dr. Jun Qi (Dana Farber Cancer Institute; original commercial source: ATCC). U251 cells were obtained from Dr. Mike Chen (City of Hope Cancer Center; original commercial source: European Collection of Authenticated Cell Cultures [ECACC]).

Authentication

1. HEK293, PANC1, and SW620 cells were directly purchased from ATCC. As a biological resource center, ATCC comprehensively performs authentication and quality-control tests on all distribution lots of cell lines using short tandem repeat (STR) profiling.
2. MDA231, H661, U251 cells were obtained from our collaborators and were not further authenticated.

Mycoplasma contamination

Plasmocin was added in all culture medium to prevent mycoplasma contamiation. All cell lines tested negative for mycoplasma contamination using a Mycoplasma PCR Detection Kit (Abm cat# G238).

Commonly misidentified lines
(See ICLAC register)

No commonly misidentified cell lines were used in this study.

Flow Cytometry

Plots

Confirm that:

☒ The axis labels state the marker and fluorochrome used (e.g. CD4-FITC).

☒ The axis scales are clearly visible. Include numbers along axes only for bottom left plot of group (a 'group' is an analysis of identical markers).

☒ All plots are contour plots with outliers or pseudocolor plots.

☒ A numerical value for number of cells or percentage (with statistics) is provided.

Methodology

Sample preparation	For competition cell culture assays, Cas9-expressing cells were transduced with the ipUSEPR (RFP+) sgRNA constructs in 96-well plates at ~50% infection. Relative RFP% refers to percentages of RFP+ cells over time after lentiviral infection, which was normalized to the RFP+% on day 0 (i.e., 48 hours after the lentiviral infection). The cell cycle was measured by Click-iT Plus EdU Alexa Fluor 647 Assay Kits (C10634, Invitrogen). Cells were exposed to 10 µM EdU at 37°C for 2 hours, and the percentage of cells in the S phase was defined by EdU-positive cells over the total singlet cells. Cellular apoptosis was detected using Annexin V Apoptosis Detection Kit (50-112-9048, Invitrogen). Live cells were defined by 4',6-diamidino-2-phenylindole (DAPI; D1306, Invitrogen) dye exclusion. The cell surface integrin αVβ5 was detected by a mouse monoclonal anti-human αVβ5 antibody (sc-13588, Santa Cruz Biotech; 1:200).
Instrument	Attune NxT flow cytometer with autosampler (ThermoFisher).
Software	Attune NxT v3.1.2 (ThermoFisher).
Cell population abundance	The RFP, αVβ5 properties were measured over the live/singlet cell population.
Gating strategy	FSC/SSC was used to get actual cells. FSC-A/FSC-H was used to get singlet. FSC/DAPI was used to gate live cells. Non-stained (or non-transduced) cells were used as negative controls for gating.

☒ Tick this box to confirm that a figure exemplifying the gating strategy is provided in the Supplementary Information.
